# Supplementary material for: MetaRibo-Seq measures translation in microbiomes
Source: Nat Commun. 2020 Jun 29;11:3268. doi: 10.1038/s41467-020-17081-z (PMC7324362; doi:10.1038/s41467-020-17081-z)
Supplement: Supplementary file 10 — Supplementary Data 7 [file 41467_2020_17081_MOESM10_ESM.zip › File2/Confidence_VeryHigh_Taxonomy/345241_out.krona.html]

Javascript must be enabled to view this page.

members
magnitude
magnitudeUnassigned
count
unassigned
taxon
rank

345241\_out

111

2
superkingdom
111

1239
phylum
111

109
186801
class

order

SRS014313\_contig\_number\_3856SRS015431\_contig\_number\_66150SRS015578\_contig\_number\_contig-100\_5608.150366SRS016495\_contig\_number\_5372SRS020869\_contig\_number\_35687SRS023914\_contig\_number\_10972SRS142503\_contig\_number\_33188SRS147022\_contig\_number\_10851SRS147139\_contig\_number\_contig-100\_1209.243338SRS147557\_contig\_number\_7370
10
109
186802

9
family
541000

1
216851
genus

1

SRS011134\_contig\_number\_contig-100\_60.301546
species
853

1
8
genus

SRS144537\_contig\_number\_18649
1263

2
2293236
species

SRS017521\_contig\_number\_23418SRS1054691\_contig\_number\_19865


SRS017103\_contig\_number\_34926SRS018351\_contig\_number\_762
species
2292370
2

species

SRS1041136\_contig\_number\_402SRS1055049\_contig\_number\_contig-100\_4023.71151SRS144183\_contig\_number\_37470
657323
3

186803
family
45

841
genus
1

1

SRS076929\_contig\_number\_15924
species
2049040

1
189330
genus

1
species

SRS104400\_contig\_number\_59420
88431

572511
genus

SRS042284\_contig\_number\_4124SRS063040\_contig\_number\_18673SRS148159\_contig\_number\_contig-100\_1884.339893SRS148196\_contig\_number\_40360
4
21

species

SRS019445\_contig\_number\_4779SRS050422\_contig\_number\_21894SRS077454\_contig\_number\_contig-100\_2332.122105SRS147346\_contig\_number\_56263
1226324
4

2
species

SRS019068\_contig\_number\_36414SRS143991\_contig\_number\_30287
765821

40520

SRS011084\_contig\_number\_23544SRS011239\_contig\_number\_2375SRS018984\_contig\_number\_19369SRS019397\_contig\_number\_5522SRS023715\_contig\_number\_28415SRS054590\_contig\_number\_contig-100\_104.29113SRS1041031\_contig\_number\_15789SRS1041130\_contig\_number\_contig-100\_2871.32664SRS142599\_contig\_number\_6304SRS146813\_contig\_number\_1686SRS147139\_contig\_number\_1591
species
11


SRS013476\_contig\_number\_42221SRS024132\_contig\_number\_52489
species
1952105
2

20
1898203
species

SRS011239\_contig\_number\_26284SRS013687\_contig\_number\_34080SRS015065\_contig\_number\_39593SRS017191\_contig\_number\_19763SRS019161\_contig\_number\_38593SRS024492\_contig\_number\_11855SRS048164\_contig\_number\_25746SRS058070\_contig\_number\_26827SRS063985\_contig\_number\_19334SRS076756\_contig\_number\_contig-100\_1213.135237SRS077024\_contig\_number\_13994SRS098073\_contig\_number\_16025SRS098571\_contig\_number\_63987SRS098881\_contig\_number\_25413SRS1041038\_contig\_number\_15340SRS1055034\_contig\_number\_contig-100\_1618.84345SRS143372\_contig\_number\_12120SRS893187\_contig\_number\_9987SRS893295\_contig\_number\_4934SRS893366\_contig\_number\_25740

45
family
31979

genus

SRS143148\_contig\_number\_contig-100\_470.150315
1
45
1485

3
species

SRS017307\_contig\_number\_contig-100\_229.181769SRS097920\_contig\_number\_contig-100\_69.192849SRS148784\_contig\_number\_27674
2293052

2
2293011
species

SRS017307\_contig\_number\_contig-100\_1002.182542SRS052027\_contig\_number\_20095

species

SRS011405\_contig\_number\_15110SRS013965\_contig\_number\_35203SRS015217\_contig\_number\_14SRS015663\_contig\_number\_24153SRS016018\_contig\_number\_contig-100\_1281.87309SRS017433\_contig\_number\_contig-100\_373.118770SRS021948\_contig\_number\_36252SRS022609\_contig\_number\_48718SRS024009\_contig\_number\_11807SRS024075\_contig\_number\_9110SRS043001\_contig\_number\_11238SRS043768\_contig\_number\_contig-100\_1709.183146SRS051031\_contig\_number\_36076SRS056259\_contig\_number\_31176SRS056273\_contig\_number\_32268SRS062701\_contig\_number\_7310SRS063190\_contig\_number\_13070SRS075078\_contig\_number\_31654SRS075341\_contig\_number\_contig-100\_193.145814SRS1041033\_contig\_number\_10007SRS1041142\_contig\_number\_2104SRS1041146\_contig\_number\_266SRS105153\_contig\_number\_4228SRS1055022\_contig\_number\_25627SRS142980\_contig\_number\_843SRS143342\_contig\_number\_1306SRS143598\_contig\_number\_23687SRS143780\_contig\_number\_31208SRS144183\_contig\_number\_contig-100\_212.197208SRS147977\_contig\_number\_11197SRS148159\_contig\_number\_38997SRS148874\_contig\_number\_8713SRS149075\_contig\_number\_16803SRS893230\_contig\_number\_12260SRS893378\_contig\_number\_18705
59620
35

2293014
species

SRS023914\_contig\_number\_23268SRS143876\_contig\_number\_18377SRS893300\_contig\_number\_8673
3

1
species

SRS050752\_contig\_number\_contig-100\_802.174180
556261

1262994
species

SRS140513\_contig\_number\_11558SRS149784\_contig\_number\_27156
2
